# Identification of single nucleotide polymorphisms in Toll-like receptor candidate genes associated with tuberculosis infection in water buffalo (*Bubalus bubalis*)

**DOI:** 10.1186/s12863-014-0139-y

**Published:** 2014-12-14

**Authors:** Flora Alfano, Simone Peletto, Maria Gabriella Lucibelli, Giorgia Borriello, Giovanna Urciuolo, Maria Grazia Maniaci, Rosanna Desiato, Michela Tarantino, Amalia Barone, Paolo Pasquali, Pier Luigi Acutis, Giorgio Galiero

**Affiliations:** Istituto Zooprofilattico Sperimentale del Mezzogiorno, Via Salute, 2, 80055, Portici, Italy; Istituto Zooprofilattico Sperimentale del Piemonte Liguria e Valle d’Aosta, Via Bologna, 148, 10154 Torino, Italy; Istituto Superiore di Sanità, Dipartimento di Sanità Pubblica Veterinaria e Sicurezza Alimentare, Viale Regina Elena, 299, 00161 Roma, Italy; Dipartimento di Agraria, Università degli Studi di Napoli “Federico II”, Via Università 100, 80055 Portici, Italy

**Keywords:** *Bubalus bubalis*, *TLRs*, Genetic resistance, Case–control study

## Abstract

**Background:**

Toll-like receptors play a key role in innate immunity by recognizing pathogens and activating appropriate responses. Pathogens express several signal molecules (pathogen-associated molecular patterns, PAMPs) essential for survival and pathogenicity. Recognition of PAMPs triggers an array of anti-microbial immune responses through the induction of various inflammatory cytokines. The objective of this work was to perform a case-control study to characterize the distribution of polymorphisms in three candidate genes (*toll-like receptor* 2, *toll-like receptor* 4, *toll-like receptor* 9) and to test their role as potential risk factors for tuberculosis infection in water buffalo (*Bubalus bubalis*).

**Results:**

The case-control study included 184 subjects, 59 of which resulted positive to both intradermal TB test and *Mycobacterium bovis* isolation (cases) and 125 resulted negative to at least three consecutive intradermal TB tests. The statistical analysis indicated that two polymorphisms exhibited significant differences in allelic frequencies between cases and controls. Indeed, the TT genotype at *TLR9* 2340 C > T locus resulted significantly associated with susceptibility to bovine tuberculosis (*P* = 0.030, OR = 3.31, 95% CI = 1.05-10.40). One polymorphism resulted significantly associated with resistance to the disease, and included the CC genotype, at the *TLR4* 672 A > C locus (*P* = 0.01, OR = 0.26, 95% CI = 0.08-0.80). Haplotype reconstruction of the *TLR2* gene revealed one haplotype (CTTACCAGCGGCCAGTCCC) associated with disease resistance (*P =* 0.04, OR = 0.51, 95% CI = 0.27–0.96), including the allelic variant associated with disease resistance.

**Conclusions:**

The work describes novel mutations in bubaline *TLR2*, *TLR4* and *TLR9* genes and presents their association with *M. bovis* infection. These results will enhance our ability to determine the risk of developing the disease by improving the knowledge of the immune mechanisms involved in host response to mycobacterial infection, and will allow the creation of multiple layers of disease resistance in herds by selective breeding.

## Background

Tuberculosis is a major zoonosis that causes serious economic losses in livestock industry. Among domestic ruminants, an economically important role, in many parts of the world and particularly in Southern Italy, is played by water buffalo (*Bubalus bubalis*). Milk from this animal species is used for the production of the worldwide famous “mozzarella di bufala” cheese, whose product specifications allow the use of raw milk [1]. It is therefore mandatory the use of milk collected from brucellosis- and tuberculosis-free herds. Tuberculosis in Southern Italy has not yet been eradicated (prevalence of 0.7% in bovine and water buffalo herds in 2012), and there is an increase of new cases of the disease (incidence of 0.65%) (data from the veterinary epidemiology monitoring field office, Istituto Zooprofilattico Sperimentale del Mezzogiorno www.izsmportici.it). The Italian National plan for tuberculosis control is based on a test-and-slaughter approach and does not allow vaccination. The genetic variation within the host may play a crucial role in the immunity to infections and resistance or susceptibility to disease, and selective breeding for disease-resistant genotypes might represent an emerging approach supporting disease control [2,3]. The role of genes in protection against bacterial infections also shown in water buffalo [4] prompted the search for polymorphisms conferring resistance to mycobacterial infection in this species.

Microbial infection initiates complex interactions between the pathogen and the host. Pathogens express several molecules, known as pathogen-associated molecular patterns (PAMPs), which are essential for survival and pathogenicity. Recognition of PAMPs triggers an array of anti-microbial immune responses through the induction of various inflammatory cytokines [5].

Toll-like receptors (*TLRs*) are reported to be involved in immune responses [6-8] and their polymorphisms have been associated with mutated susceptibility to mycobacteria, the causative agent of bovine tuberculosis, both in humans and different animal species [9-15]. *TLRs* are transmembrane proteins that play a key role in innate immunity by recognizing pathogens and subsequently activating appropriate responses. They are characterized by an extracellular N-terminal domain constituted by 16 to 28 leucine rich repeats (LRR) involved in ligand recognition [16], and an intracellular C-terminal domain known as the toll/IL-1 receptor (TIR) domain, required for the interaction and recruitment of various adaptor molecules to activate the downstream signalling pathways [17]. In water buffalo, each cell contains 25 chromosome pairs (*Bubalus bubalis* 2n = 50) [18], and the *TLRs* 2 and 9 loci are localized on chromosomes 17 and 21, respectively, while the *TLR* 4 locus is located on chromosome 3 [19].

Toll-like receptors 2 and 4 mediate their effects by recognizing bacteria, including mycobacteria. Toll-like receptor 2 is sensitive to peptidoglycan and to lipoarabinomannan. Toll-like receptor 4 is involved in bacterial lipopolysaccharide recognition [20], but its sensitivity to mycobacterial antigens has also been reported [21]. Toll-like receptor 9 is essential for responses to bacterial DNA and in particular to unmethylated CpG dinucleotides (CpG DNA) [22].

The aim of this study was (i) to identify single nucleotide polymorphisms (SNPs) in the coding sequence (CDS) of three candidate genes: *toll-like receptor 2* (*TLR 2*)*, toll-like receptor 4* (*TLR 4*)*, toll-like receptor 9* (*TLR 9*), (ii) to characterize the distribution of the detected polymorphisms and (iii) to perform a case-control study to test their role as potential risk factors for tuberculosis infection in water buffalo.

## Results

### Identification of SNPs

The water buffalo *TLR2* and *TLR9* genes consist of two exons, while the *TLR4* consists of three exons. The analysis of the entire CDSs enabled us to identify 29 new SNPs. Specifically, 18 were identified in *TLR*2 (five non-synonymous and 13 synonymous), nine in *TLR*4 (five non-synonymous and four synonymous), and two in *TLR9* (both synonymous). All the found SNPs were bi-allelic with the exception of a tri-allelic SNP (572 A > CG) found in *TLR4.* In addition we identified a dinucleotidic SNP (482/483 GC > CT) in *TLR2.* Table 1 shows all the SNPs, their positions in the CDS, the encoded protein codon and the GenBank reference number.

**Table 1:** **Detected SNPs in bubaline** ***TLRs2*** **,** ***4*** **and** ***9*** **genes**

**Gene**	**SNP** ^***a***^	**AA change** ^***b***^	**Protein domain** ^***c***^	**dbSNP ID**
*TLR2*	42 C > T	Silent 14	-^*d*^	rs1388116474:C > T
	53 C > T	M18T	-	rs1388116475:C > T
	108 C > T	Silent 36	LRR 8	rs1388116476:C > T
	153 G > A	Silent 51	LRR_RI	rs1388116477:G > A
	156 C > T	Silent 52	LRR_RI	rs1388116478:C > T
	374 T > C	A125V	LRR 8	rs1388116479:T > C
	381 A > G	Silent 127	LRR 8	rs1388116480:A > G
	482/483 GC > CT	S161T	LRR 8	rs1388116481:GC > CT
	519 G > C	Silent 173	LRR_RI	rs1388116482:G > C
	1034 A > G	S345N	-	rs1388116483:A > G
	1375 T > C	Silent 459	LRR4	rs1388116484:T > C
	1407 C > T	Silent 469	LRR4	rs1388116485:C > T
	1650 G > A	Silent 550	LRR_CT	rs1388116486:G > A
	1678 A > G	A560T	LRR_CT	rs1388116487:A > G
	1707 C > T^*e*^	Silent 569	LRR_CT	rs1388116488:C > T
	1731 C > T	Silent 577	LRR_CT	rs1388116489:C > T
	1740 C > T	Silent 580	LRR_CT	rs1388116490:C > T
	2064 T > C	Silent 688	TIR	rs1388116491:T > C
*TLR4*	572 A > C^*f*^	Y191S	LRR8	rs1388116492:A > CG
	572 A > G^*f*^	silent	LRR8	rs1388116492:A > CG
	574 C > T	Q192W	LRR8	rs1388116493:C > T
	575 A > G	Q192W	LRR8	rs1388116494:A > G
	576 T > G	Silent192	LRR8	rs1388116495:T > G
	577 G > A	E193K	LRR8	rs1388116496:G > A
	579 A > G	Silent193	LRR8	rs1388116497:A > G
	647 G > A	Silent216	LRR_RI	rs1388116498:G > A
	662 G > A	Silent221	-	rs1388116499:G > A
	672 A > C	Silent224	-	rs1388116500:A > C
*TLR9*	2340 C > T	Silent780	-	rs1388116501:C > T
	2475 A > G	Silent825	-	rs1388116502:A > G

^*a*^SNPs positions were calculated taking the ATG start codon as position 1 based on the sequences GenBank: HM756161 (*TLR2* gene), GenBank: JN786600 (*TLR4* gene), GenBank: HQ242778 (*TLR9* gene).

^*b*^Amino acid positions are given according to the ATG start codon.

^*c*^Protein domain has been predicted from the cds sequence by using the Conserved Domain Database available at http://www.ncbi.nlm.nih.gov/Structure/cdd/wrpsb.cgi [23,24].

^*d*^Protein position with unknown function.

^*e*^This SNP is conserved also in the bovine species [9].

^*f*^This polymorphic site exhibits a SNP with three different alleles (572 A > CG).

### Case-control study

Calculation of the allelic and genotypic frequencies, and application of the χ2 test, revealed that both populations (cases and controls) conformed to the Hardy-Weinberg equilibrium with regard to all the polymorphic loci (*P* > 0.05) of the *TLRs 2, 4* and *9* genes.

As a first step of the genetic association study, Pearson’s chi-squared test was carried out for each SNP both independently and as genotype. As many SNPs exhibited a strong linkage disequilibrium a multiple testing correction was applied by permutation analysis. Within the 29 analysed polymorphic sites, three SNPs exhibited statistically significant differences in frequency distribution of one or more associated genotypes between cases and controls (Table 2). In particular, one SNP was located in the *TLR2* gene (1650 G > A ), one SNP in *TLR4* (672 A > C) and one in *TLR9* (2340 C > T). Then, in order to test inheritance model, a deviation test from additivity was performed with plink version 1.07, suggesting in our case an additive model of inheritance. SNPs significantly associated to the disease (*P* < 0.05) were used for further analysis: a logistic regression model was performed to calculate the Odds Ratio (OR) with the wild type category used as a baseline (Table 3).

**Table 2:** **Results of permutation analysis**

**Chromosome**	**SNP**	**Empirical** ***P*** **-value**
17	42-C > T	0.9000
17	53-C > T	0.1967
17	108-C > T	0.1714
17	153-G > A	0.2195
17	156-C > T	0.9452
17	374-T > C	0.1229
17	381-A > G	0.9788
17	482/483-GC > CT	0.8222
17	519-G > C	0.5175
17	1034-A > G	0.2191
17	1375-T > C	0.1302
17	1407-C > T	0.8738
17	1650-G > A	0.0138*
17	1678-A > G	0.1505
17	1707-C > T	0.2203
17	1731-C > T	0.9286
17	1740-C > T	0.8985
17	2064-T > C	0.1296
3	572 C > A G	0.4909
3	574 C > T	0.4288
3	575 A > G	0.4380
3	576 T > G	0.2490
3	577 G > A	0.6373
3	579 A > G	0.2551
3	647 G > A	0.1735
3	662 G > A	0.1006
3	672 A > C	0.0183*
21	2340 C > T	0.0521*
21	2475 A > G	0.1254

*statistically significant.

**Table 3:** **Polymorphic sites including genotypes with statistically significant differences in frequency distribution between cases and controls**

**Gene**	**SNPs**	**Genotype**	***P*** **-value**	**OR**	**CI 95%**
TLR2	1650 G > A	G/A	0.166	0.612	0.26 - 1.01
		A/A	0.021	0.000	--
TLR4	672 A > C	A/C	0.689	0.865	0.42 - 1.76
		**C/C**	**0.012**	**0.258**	**0.08 - 0.80**
TLR9	2340 C > T	C/T	0.048	2.917	0.96 - 8.89
		**T/T**	**0.030**	**3.306**	**1.05 - 10.40**

Bold lines highlight genotypes with statistically significant *P*-values and ORs.

In the *TLR2* gene, the 1650 G > A locus exhibited a χ2 test of independence with a significant *P-*value (*P* = 0.018) while the OR for the AA genotype, calculated with the Woolf’s method, exhibited a not statistically significant value (*P* = 0.0512, OR = 0.10, 95% CI = 0.01-1.74). However, in this case, a significant test for trend (*P* = 0.01) in calculating OR values in logistic regression indicates a plausible strong correlation with resistance to the disease.

For the *TLR4* gene, the SNP 672 A > C showed a χ2 test with a significant *P*-value (*P* = 0.031); the OR demonstrated a significant *P-*value (*P* = 0.012) for the CC genotype at the 672 A > C locus and a value lower than 1 (OR = 0.26, 95% CI = 0.08 to 0.80), thus indicating a significant association with resistance to tuberculosis.

In the *TLR*9 gene, the analysis revealed one polymorphic site (2340 C > T) with a significant *P*-value (0.053) at the χ2 test including one genotype (T/T) associated with susceptibility to the disease (*P* = 0.030 and OR = 3.31, 95% CI = 1.05-10.40).

Haplotype reconstruction based on *TLR2* polymorphisms performed by PHASE software, generated 25 possible haplotypes, seven of which displayed a frequency greater than 0.01% in the whole sample. The software PHASE can reveal any significant imbalance in haplotype distribution between groups by performing a permutation test; the application of this analysis to the detected haplotypes revealed the presence of one haplotype (CTTACCAGCGGCCAGTCCC) associated with disease resistance (*P* = 0.041 and OR = 0.51, 95% CI = 0.27-0.96). This haplotype contained the allelic variant that resulted possibly associated with resistance to the disease.

In the *TLR4* gene, 34 haplotypes were observed in the two tested groups, six of which displayed a frequency greater than 0.01% in the whole population. None of the predicted haplotypes showed a significant association with resistance/susceptibility to tuberculosis.

Finally, four haplotypes were observed in the *TLR9* gene, none of which displayed a significant association with resistance/susceptibility to tuberculosis.

## Discussion

The polymorphisms of single nucleotides constitute excellent genetic markers for various population studies as they can, for instance, reveal traces of natural selection. Moreover, it is now known that the contribution of genes to the incidence of or predisposition to diseases can be determined by comparing inter-individual genetic differences.

Each individual, to a greater or lesser degree than others, is genetically prone to develop certain diseases. In most cases, the susceptibility or resistance to a given pathology does not of course mean that the individual will necessarily be affected by the disease or remain disease-free; it does, however, indicate a higher or lower risk than that of the general population. Indeed, the onset of symptoms is due to an interaction between genetic factors and environmental factors. Nevertheless, cases have been described in which genetic factors are sufficient to determine the course of infection. In scrapie, for instance, which is a transmissible disease of sheep and goats caused by a prion, there are reports of genotypes that are defined as “resistant”, which are practically refractory to natural infection [25,26].

Our analysis of the water buffalo *TLR2*, *TLR4* and *TLR9* genes enabled us to identify 29 SNPs. In the three genes, the 76% of the SNPs fell within the LRR protein domains, regions responsible for ligand recognition. This finding is in agreement with conservation of the transmembrane and functional TIR domains already observed in human, bovine and murine *TLRs* genes [9-13].

The case-control study carried out revealed the presence of associations between some of the identified polymorphisms and resistance/susceptibility to the disease caused by *M. bovis*. Specifically, in *TLR*4, we observed a correlation between resistance to disease and the genotype C/C at the 672 A > C locus (*P* = 0.012, OR = 0.26, 95% CI = 0.08-0.80) when a logistic regression model was performed. A likewise correlation with weak statistical evidence was seen in *TLR2* at the 1650 A > G locus when a logistic regression model was performed between wild type genotype and mutated genotype (*P* = 0.0512, OR = 0.10, 95% CI = 0.01-1.74). Moreover, a correlation was seen between susceptibility to tuberculosis and the T/T genotype at the 2340 C > T locus in the *TLR9* gene (*P* = 0.030, OR = 3.31, 95% CI = 1.05-10.40).

Haplotype reconstruction based on *TLR2* polymorphisms enabled us to ascertain whether the interaction of the single alleles exerted an influence on the animal’s response to the disease; indeed, this analysis revealed a haplotype (CTTACCAGCGGCCAGTCCC) that was associated with disease resistance (*P* = 0.041 and OR = 0.51, 95% CI = 0.27-0.96). This haplotype contained the allelic variant which resulted possibly associated with resistance to the disease. However, as this variant is not exclusive of the associated haplotype, we may hypothesise that other loci exert a modulatory effect.

Haplotype reconstruction and the permutation test conducted on the polymorphisms identified in *TLR4* did not retrieve any disease-associated haplotype.

Previous studies have demonstrated the influence of genetic factors and some gene polymorphisms on susceptibility to several bacterial diseases, including TB. TLRs have been found to be associated with both the innate immune and adaptive immune responses, and play a crucial role in the immune recognition of *M. tuberculosis.* Mutations and polymorphisms in TLRs and TLR signalling molecules have revealed the importance of TLRs both in human and animal defence against disease [14-22,25-28]. One nsSNP in *TLR2* (Arg753Gln) has been shown to increase human predisposition to staphylococcal infection, tuberculosis, rheumatic fever and urinary tract infection [10-15]. A nsSNP in the bovine *TLR2* gene (Val220Met) has been shown to decrease the response to *M. avium* subsp*. paratuberculosis* (Map) [11], while another SNP in the same gene, C1903T (Silent569), was found to be associated with resistance to Map infection in cattle [9]. Several polymorphisms have been found in the bovine *TLR4* gene, 12 SNPs affecting ligand binding domain [29] and four SNPs associated with susceptibility to Map infection [11]. Studies conducted on the bovine *TLR9* gene demonstrated a high degree of genetic variability in this gene [14-22,25-30], even though no significant association with bovine tuberculosis could be inferred from mutations in this gene [14]. A SNP in the human *TLR9* gene (rs352139) was found to be associated with susceptibility to TB (*P* = 0.01, OR = 2.37) [31]. In our study, among the polymorphisms identified in water buffalo, only one (C1707T in the *TLR2* gene) showed correspondence to a polymorphism reported in cattle (T1903C) [9]. Specifically, Koets and coworkers [9] found this SNP associated with resistance to Map, while in water buffalo the same polymorphism did not show any significant association with susceptibility/resistance to TB.

To date, it is estimated that about 50 human diseases are caused entirely or partly by synonymous mutations, and it has also been demonstrated that they play a role in disease resistance [32,33]. In the human *TLR*2, for example, synonymous mutations have been identified which are associated with resistance to liver cell carcinoma [17]. A recent meta-analysis identified a significant association between an intron mutation of *TLR*9 and tuberculosis in humans [34].

Moreover, it has been observed that silent mutations can interfere with various stages of protein “construction”, from DNA transcription to the translation of mRNA into protein.

Silent changes in codons located in the exonic splicing enhancers (ESE) can prevent introns from being properly eliminated. A notable example of the damage that can be caused by mutations in a splicing enhancer has been documented for the *CFTR* (Cystic Fibrosis Transmembrane Conductance Regulator) gene [35,36].

Finally, synonymous mutations can alter the stability of the transcript by reducing its affinity for RNA binding proteins. Indeed, in humans, a synonymous mutation of the gene coding for corneodesmosine (*CDSN*) has been associated with the onset of psoriasis in various ethnic groups [37].

An alternative hypothesis could be that some of the detected synonymous mutations are not directly related to disease susceptibility/resistance, but rather that one or more are linked to other functional mutations on the same chromosome which have not yet been identified. Functional studies will be crucial in order to understand how these polymorphisms act.

## Conclusions

The work describes novel mutations in the bubaline *TLR2*, *TLR4* and *TLR9* genes and describes their association with *M. bovis* infection. Studies of the genetic factors involved in complex diseases have not yet provided clear explanations for the onset of such diseases, though they may help to identify their underlying physio-pathological pathways. A thorough knowledge of these mechanisms will improve our ability to determine the risk of developing the disease and to understand it in its entirety. This will pave the way to create multiple layers of disease resistance in herds by selective breeding and also to identify and synthesize innovative drugs.

## Methods

### Study design

Genetic association between SNPs and TB was studied by a case-control approach on 184 water buffaloes from 22 herds in the Campania region. Sample collection was performed within the Italian National Plan for the control of tuberculosis, which imposes culling of all the subjects positive to the delayed hypersensitivity test [38] and microbiological processing of the lymphoid tissue for isolation of *M. bovis* [38]. Our analyses, performed during the period 2009-2011, revealed the presence of 59 animals positive to both intradermal TB test and *M. bovis* microbiological isolation. These 59 animals were therefore classified as cases for the case-control study. The choice of the microbiological test to confirm cases identification was considered in order to exclude the possibility of false-positive subjects diagnosed by the delayed hypersensitivity test. The control subjects were collected among the animals which tested negative to repeated intradermal TB tests (at least three tests) during the entire period of the study. The control animals were at least five years-old, and were born and raised in the same herds of cases to ensure an equal level of exposure to infection. The number of controls was estimated taking into account an odds ratio value of 2.0 as a threshold for significance, with a ratio for cases and controls of 1:2 (power = 80%, confidence = 95%) [12]. Based on these parameters, with an imposed number of cases (59), the required number of controls was 108 for a one-tailed analysis. The present case-control study therefore included 59 cases and 125 controls.

### TLRs sequencing

DNA was extracted from blood (control animals) and lymph node (cases) samples using the QIAamp DNA mini kit (Qiagen, Hilden, Germany) according to the manufacturer’s protocol. PCR primers for amplification of the CDS of the genes *TLR2*, *TLR4* and *TLR9* were designed using the Web interface Primer3; they are shown in Table 4. The *TLR2* coding sequence was amplified using two primer pairs according to the following touchdown PCR protocol: initial step of 7 cycles of 1 min at 95°C, 1 min at 65°C (decreasing by 1°C after each cycle), 1 min 30 s at 72°C, followed by 35 cycles of 1 min at 95°C, 1 min at 58°C, 1 min 30 s with a final extension of 10 min at 72°C. The *TLR4* CDS was amplified using four primers sets with the following thermal profile: 35 cycles of 30 s at 95°C, 30 s at T_ann_ and 45 s at 72°C, with a final extension of 10 min at 72°C. T_ann_ is the annealing temperature specifically indicated for each primers pair in Table 4. The *TLR9* CDS was amplified using six primer sets with the following thermal profile: 5 cycles of 30 s at 95°C, 20 s at 60°C, and 15 s at 72°C followed by 30 additional cycles of 20 s at 95°C, 20 s at T_ann_, and 30 s at 72°C, with a final extension of 10 min at 72°C. All thermal profiles included an initial step at 95°C for 15 min for Taq DNA polymerase activation. PCR reactions were performed with the HotStar HiFidelity Polymerase Kit (Qiagen), and the reaction mixture included 50 ng of genomic DNA and 0.5 μM of each primer (forward and reverse) in a final volume of 25 μl. Amplicons were purified (QiaQuick purification kit, Qiagen), bi-directionally sequenced using the Big Dye Terminator cycle sequencing kit v.1.1 or v.3.1 (Life Technologies) and purified using the DyeEx spin kit (Qiagen). Samples (5μl) were denatured with 10 μl of Hi-Di formamide (Life Technologies) at 95°C for 5 min, and separated by capillary electrophoresis on either ABI PRISM 310 or 3130 sequencers (Life Technologies). Sequencing data were manually inspected by the Sequencing Analysis software v5.4 (Life Technologies).

**Table 4:** **PCR primers, annealing temperatures and amplicon length of amplified bubaline** ***TLRs*** **2, 4 and 9 genes**

**Oligo**	**Sequence (5′ 3′)**	**PCR T**_**ann**_ **(°C)**	**Amplicon (bp)**
TLR2 A	For: TTTGTAGGTCAAATCACTGGACA	58^*a*^	1331 bp
	Rev: TCCTGGCCACTGACAAGTTT		
TLR2 B	For: GCCCTTCCTTCAAACCTTG	58^*a*^	1234 bp
	Rev: CACCACCAGACCAAGACTGA		
TLR4 A	For: GTGTGGAGACCTAGATGACTGG	60	706 bp
	Rev: GTACGCTATCCGGAATTGTTCA		
TLR4 B	For: CTTTCCTGGAGGGACTGTGC	60	435 bp
	Rev: CCACGAAGTTTGAACCTAAGGTAA		
TLR4 C	For: CTACCAAGCCTTCAGTATCTAG	60	743 bp
	Rev: GGCATGTCCTCCATATCTAAAG		
TLR4 D	For: AAGGACCAGAGGCAGCTCTT	58	801 bp
	Rev: TAACTGAACACGCCCTGCAT		
TLR9 A	For: CCAGCCTCTCCTTAATCTCC	54	718 bp
	Rev: CGGAACCAATCTTTCTCTAGTT		
TLR9 B	For: CCTGACACCTTCAGTCACCT	55	651 bp
	Rev: GCGGGTAAACATCTCTTGCT		
TLR9 C	For: CGTCAGCTCAAAGGACTTCA	56	546 bp
	Rev: AGGGTGTGCAGATGGTTCTC		
TLR9 D	For: GGGAGACCTCTATCTCTGCTTT	56	428 bp
	Rev: CGCTCACGTCTAGGATTTTC		
TLR9 E	For: CTTCAGAAGCTGGACGTGAG	55	685 bp
	Rev: TCTTGCGGCTGCTGTAGAC		
TLR9 F	For: TGCTCTATGATGCCTTCGTG	55	424 bp
	Rev: AGGTTGGCCCAGAAACTACC		

^*a*^For these primer pairs a touchdown PCR thermal profile was used.

### SNP selection and genotyping

Sequences were analyzed by multiple alignment using BioEdit v.7.1.3 [39] and SeqManII 5.00 software (DNASTAR Inc.). The 184 water buffalo sequences of the *TLR2*, *TLR4* and *TLR9* genes were compared with the publicly available sequences GenBank:HM756161 (*Toll-like receptor 2 gene,* complete cds), GenBank:JN786600 (*Toll-like receptor 4 gene,* complete cds), GenBank:HQ242778 (*Toll-like receptor 9 gene,* complete cds), respectively. Each forward and reverse sequence from a single DNA sample was compared with the other in order to generate a consensus sequence and to identify polymorphisms among the samples.

### Genetic association analysis

Genotype frequencies were tested for deviation from Hardy-Weinberg equilibrium (HWE) by using Fisher Exact test and Chi-square test (χ2). Pearson’s χ2 test was carried out for each SNP both independently and by using a multiple testing correction based on permutation [40,41].

SNPs that were significantly associated with the disease were entered in an univariate logistic regression model. All statistical analyses were performed using Stata statistical software, release 10.0 (Stata Corp., College Station, TX, USA) and PLINK software (PLINK 1.07, Shaun Purcell http://pngu.mgh.harvard.edu/purcell/plink/).

Haplotype reconstruction was performed using PHASE software, version 2.1 [42].
